# Comparing the effect of transcutaneous electrical nerve stimulation and massage therapy on post laparoscopic shoulder pain: a randomized clinical trial

**DOI:** 10.1186/s12891-023-06905-w

**Published:** 2023-09-28

**Authors:** Mobin Mottahedi, MohammadBagher Shamsi, Samira Fatahi Babani, Shahrbanoo Goli, Parisa Rizevandi

**Affiliations:** 1 Department of Operating Room, School of Paramedical, Kermanshah University of Medical Sciences, Kermanshah, Iran; 2 Department of Physiotherapy, School of Rehabilitation Sciences, Kermanshah University of Medical Sciences, Kermanshah, Iran; 3 Department of Epidemiology, School of Public Health, Shahroud University of Medical Sciences, Shahroud, Iran; 4 Department of Operating Room, School of Paramedical Sciences, Kermanshah University of Medical Sciences, Kermanshah, Iran

**Keywords:** Shoulder pain, Laparoscopic cholecystectomy, Massage therapy, Transcutaneous electrical nerve stimulation (TENS)

## Abstract

**Background:**

Shoulder pain is a common clinical problem after laparoscopic surgeries. The use of non-pharmacological massage and transcutaneous electrical nerve stimulation (TENS) as an adjunct to routine treatment is increasing to provide optimal pain relief. Therefore, we aimed to determine the effect of TENS and massage therapy on post laparoscopic shoulder pain (PLSP).

**Methods:**

This study was conducted on 138 patients who underwent laparoscopic cholecystectomy. Patients were randomly divided into three groups: massage plus conventional pharmacological treatment (n = 46), TENS plus conventional pharmacological treatment (n = 46), and conventional pharmacological treatment (n = 46). Massage and TENS were performed three consecutive times after the patients regained consciousness in the inpatient wards. The intensity of Shoulder pain was evaluated using a visual analog scale before and 20 min after each treatment.

**Results:**

Both massage therapy and TENS led to a significant reduction in the intensity of PLPS compared to the control group in all three measured times (p < 0.001). However, no significant difference was observed between TENS and massage at any of the three-time points.

**Conclusions:**

This study’s findings demonstrated that massage and TENS techniques could reduce PLSP.

**Trial registration:**

Registered in the Iranian registry of clinical trials (www.irct.ir) in 05/02/2022 with the following code: IRCT20200206046395N1.

## Introduction

Laparoscopic cholecystectomy is the standard procedure in gallbladder surgery [1]. Although laparoscopy has optimal results compared to open surgery, such as cosmetic effects, rapid discharge, and less pain at the incision site, post laparoscopic shoulder pain (PLSP) is one of the most common complications [2]. The prevalence of PLSP varies from 30 to 90%, and this relatively high rate is a challenging issue [3]. Although the underlying cause of PLSP is unknown, tissue manipulation, diaphragmatic pressure due to pneumoperitoneum, and carbonic acid production are the possible causes [4].

Various approaches have been investigated to relieve PLSP following laparoscopic cholecystectomy. In some studies, different surgical techniques, drainage, respiratory approaches [5], drugs such as lidocaine [2], duloxetine [6] ,and clonidine [7] were used. Despite few studies, they are limited, indefinite, and have controversial results.

Multifaceted methods for pain relief are essential due to the complex pain mechanism after laparoscopic cholecystectomy [8]. Non-pharmacological methods used for these purposes include: Acupuncture, music, rhythmic breathing, relaxation, meditation, TENS ,and massage [9].

“Therapeutic massage is the manipulation of the soft tissue of whole body areas to bring about generalized improvements in health, such as relaxation or improved sleep, or specific physical benefits, such as relief of muscular aches and pains” [10]. It is a non-invasive method with an easy application used in various clinical centers to increase the quality of surgery [11]. The benefits of massage in pain relief are supported by multiple studies on colorectal surgery [12], coronary artery bypass grafting [13], cesarean Sect. [14], and cholecystectomy [15].

In addition, transcutaneous electrical nerve stimulation (TENS) as an essential strategy in interventions and clinical approaches for analgesia and its side effects due to unique benefits such as ease of use, efficiency, inexpensiveness, and low risk is increasing. TENS uses batteries and electrodes on the skin based on the adjusted frequency and pulse width by providing alternating current for various therapeutic purposes [16]. TENS has been instrumental in postoperative analgesia in many hernia repair surgeries [17], arthroscopy [18], gynecological laparoscopy [19], and liposuction [20].

Although various techniques have significant effects in reducing PLSP, to our knowledge, no previous research compared these two interventions, massage therapy and TENS, for the relief of shoulder pain after laparoscopic cholecystectomy. Considering the prevalence of 63% of shoulder pain after laparoscopic cholecystectomy and the increase of patients who complain of PLSP [21], it is necessary to ensure adequate pain management. Because insufficient and poor management leads to delayed recovery, adverse clinical outcomes and increased treatment costs [22]. Therefore, we aimed to determine the effect of TENS and massage therapy on PLSP. We hypothesized that massage and TENS could reduce PLSP in the first hours after cholecystectomy.

## Materials and methods

### Design

This study is a prospective, randomized, single-blind, parallel controlled, and triple-arm clinical trial. It was conducted on 138 patients with gallstones treated with laparoscopic cholecystectomy at Imam Reza Hospital, affiliated with Kermanshah University of Medical Sciences, Iran. Eligible patients were selected from February 2022 to August 2022. They were randomly divided into three groups, including massage (A), TENS (B), and control (C). The ethics committee of the Vice-Chancellor of Research approved the study at Kermanshah University of Medical Sciences (Code: IR.KUMS.REC.1400.757). It was registered on the Iranian Clinical trial Registry with identification number IRCT IRCT20200206046395N1 (registration date: 05/02/2022).

### Inclusion and exclusion criteria

The inclusion criteria for patients were:

- Undergoing laparoscopic cholecystectomy surgery.
- Visual Analogue Scale (VAS) shoulder pain score at baseline (within 3 h after surgery) ≥ to 3.
- Class I or II physical conditions based on the American Society of Anesthesiology (ASA).
- No history of mental, motor ,or cognitive disorders according to medical records.
- Lack of shoulder pain before surgery.

The exclusion criteria were:

- Age under 18 years and over 60 years.
- Addiction to painkillers and sedatives.
- Ulcers, phlebitis, trauma ,and arthritis in the massage area.
- Contraindications to TENS such as pregnancy and internal pacemaker.

### Sample size calculation

**Fig. 1:**
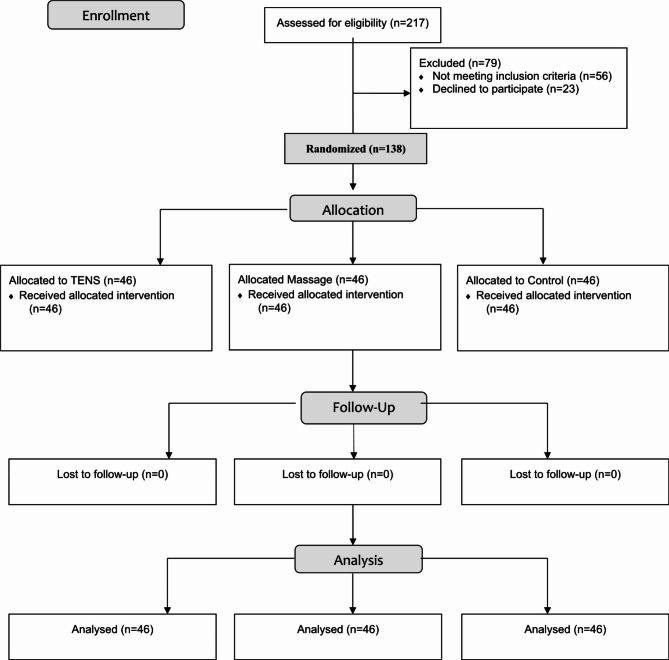
CONSORT flow diagram

After a pilot study on 30 participants (10 in each group), the minimum number of samples based on a 10-point pain scale 4 h after cholecystectomy using the mean and standard deviation (3.83 ± 1.94 in the control group and 5 ± 1.67 in the intervention group) was identified. By accepting α = 0.05 and a power of 80%, the standard sample size for each group was calculated as 38 patients (114 in total). Considering a 20% drop rate, 46 participants were included in each group (138 patients in total) (Fig. 1).

### Randomization and blinding

After evaluating the admitted patients based on the inclusion and exclusion criteria and completing the informed consent form, they were placed in massage, TENS, or control groups using a random block size of 6. A randomization sequence was performed using a random number generator.

Twenty-three opaque envelopes and 138 cards were created for the random allocation concealment process. The envelopes were opened simultaneously as the individuals were assigned to the groups. Before random sampling, the researcher and participant did not know each individual would belong to which group. Concealment was also observed using this method. Participants were aware of the intervention, but the data collector and statistical analyst were unaware. In addition, the form related to the baseline assessments was initially completed before assigning the person to groups. The participants were separated and had no contact to prevent bias.

### Pain assessment

Patients were asked to rate their pain in the shoulder area by marking a cross on a 10 cm horizontal VAS. It is one of the standard tools for measuring pain in studies with a clinical approach. In the study of Al-Ghadir et al. [23], the reliability of this tool for pain by the intra-class correlation coefficient was reported 0.97, which can be confirmed as one of the most reliable tools for measuring pain. Zero indicated no pain, and ten showed unbearable pain on this scale. Pain intensity in this study was categorized into four groups, 0 = painless, 1 to 3 = mild, 4 to 7 = moderate, and 8 to 10 = severe pain.

### Intervention

On the morning of surgery, each patient was asked to rate pain VAS using understandable and straightforward sentences during a training session. After transferring patients to the postoperative ward and consciousness recovery, participants in the intervention group received TENS or massage in addition to conventional pharmacological treatment. No intervention was applied to the control group, who only received conventional pharmacological treatment. Acetaminophen (Paracetamol) was prescribed 15 mg/kg/dose every 8 h according to our standard organizational protocol to relieve postoperative pain. In addition, patients received intramuscular Pethidine 25 mg/mL at regular intervals in case of more severe pain.

Both massage therapy techniques and TENS were performed at regular intervals of 4, 8, and 12 h after surgery by a trained individual and under the supervision of a physiotherapist. The interventions lasted 30 min in a quiet environment away from noise (3 times, 90 min in total).

In addition, to avoid bias, in all groups, the researcher attended during the study at the mentioned times, and asked the participants to lie on the bed for 30 min in a supine position with their head at a height of 30 Degree headrest.

#### Massage

**Fig. 2:**
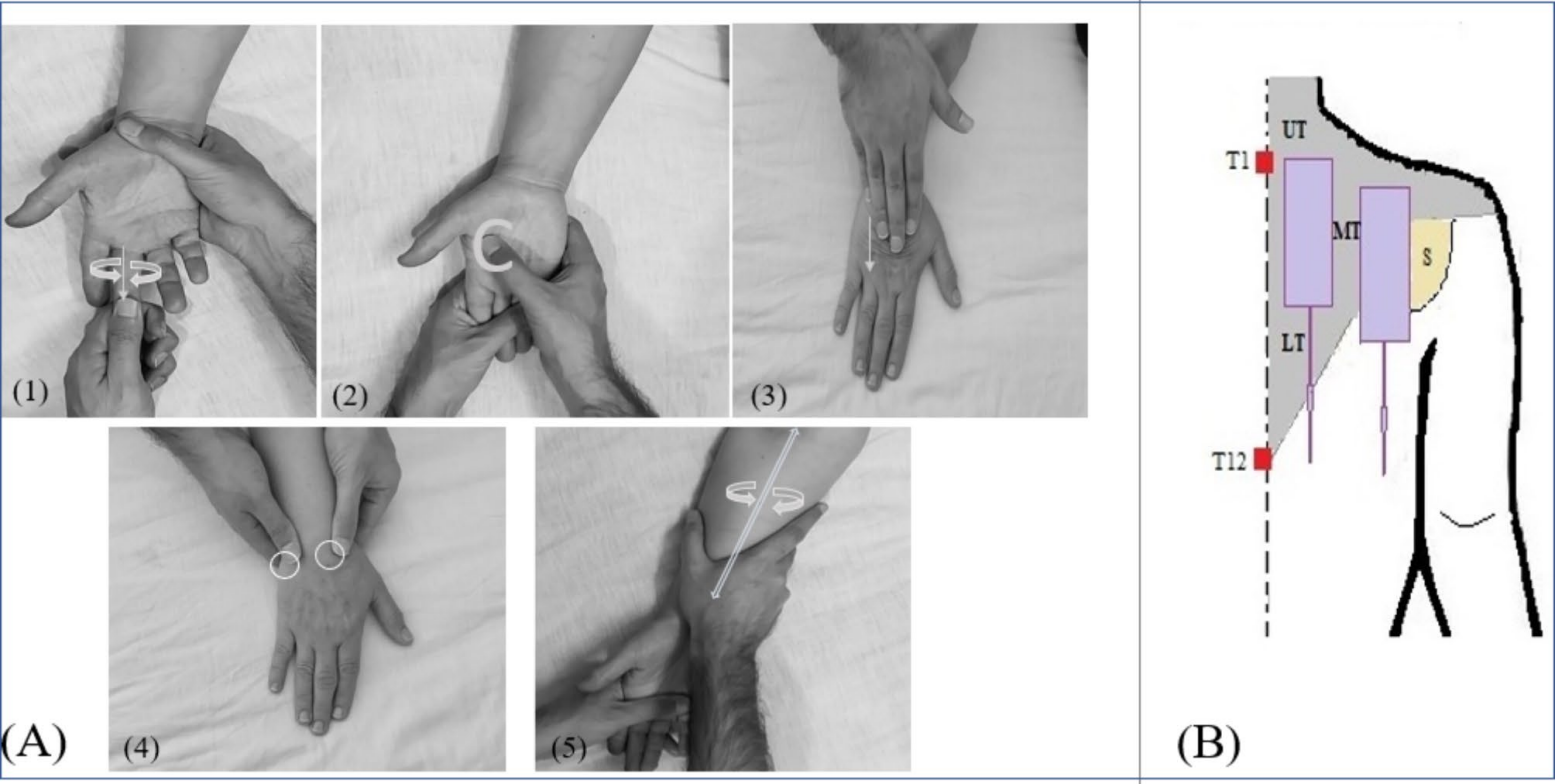
This figure shows the areas of interventions. (**A**) Massage after PLSP (1). finger massage, (2) palm massage, (3) back hand massage, (4) wrist massage, (5) forearm massage. (**B**) TENS after PLSP. Gray indicates Trapezius muscle. UT = Upper Trapezius, MT = Middle Trapezius, LT = Lower Trapezius. The Scapula is represented in Light yellow

The massage was performed in the first intervention group using this protocol:

The masseur washed his hands thoroughly and dried them with a towel. He applied massage on the patient’s dominant hand using kneading-friction-petrissage techniques. The wrist was held in one hand, and with the other hand, the massage started with direct pressure from the base of each finger and circularly continued to the tip. The palm was kneaded with C-like movements and firm pressure in alternating directions, then the back of the hand was stretched with forward movements from the wrist to the base of each finger. Around the wrist bones, the massage continued with circular movements of the thumbs clockwise and then counterclockwise. While the patient’s arm was lying flat on the bed, the therapist’s palm was used for upward movements from the wrist to below the elbow and downward movements from the elbow to the wrist. Finally, the massage ended with gentle twisting movements of the forearm (Fig. 2). Patients were asked to inform the researcher having any risk factor to avoid any problem during the massage therapy. After the first set of massage and to ensure the patient’s comfort, the second set of massage was performed similarly, without using any special equipment on the non-dominant hand. The massage procedure was followed using a coherent program and continued with the appropriate rhythm and pressure.

Petrissage Massage: Apply direct pressure slowly and rhythmically with the fingertips.

Friction Massage: Rubbing in small areas in a circle where the masseur presses the area with the front of the fingertips or the front of the palm.

Kneading Massage: It is similar to twisting and changes the compression direction in succession [24].

#### TENS

Patients in the second intervention group received TENS. Initially, the researcher watched the skin of the scapula and shoulders to ensure that no foreign objects obstructed the attachment of four electrodes (5 cm x 10 cm). Then the electrodes were placed on the skin in the area of trapezius muscle and scapula, in both sides of the shoulders, parallel to the spine (Fig. 2). After placing the self-adhesive electrodes at a distance of 5 cm in areas, a TENS current with a frequency of 150 Hz and a pulse width of 75 microseconds [25] was applied. During the intervention, the intensity of the TENS device was constantly increased by the researcher up to the extent that the maximum effect of the tingling sense did not cause discomfort and muscle contraction (the maximum sensory threshold for pain).

### Data collection

Participants’ data, including demographic and clinical characteristics, were collected using two parts form and multiple-choice questions.

In Its first part, age, sex, education and previous history of abdominal surgery were recorded one day before the operation.

In the second part, duration of surgery, duration of anesthesia, amount of analgesia used during the intervention and severity of PLSP were assessed by the outcome assessor, unaware of the type of interventions and randomization. According to the beginning of the first, second and third interventions at 4, 8 and 12 h after cholecystectomy, pain was recorded six times. T_1_: 20 min before the start of the first intervention, T_2_: 20 min after the first intervention, T_3_: 20 min before the start of the second intervention, T_4_: 20 min after the second intervention, T_5_: 20 min before the start of the third intervention, T_6_: 20 min after the third intervention.

### Statistical methods

Data analysis was conducted using the SPSS 25.0 software. In the present study, Data normality was confirmed using the Kolmogorov-Smirnov. ANOVA analysis of variance compared quantitative variables in three groups. Also, the variables of gender, education, and history of previous abdominal surgery were evaluated with the Chi-square test. In each group, ANOVA with repeated measures was used to compare the trend of changes in the severity of PLSP at 4, 8, and 12 h after surgery. P-value < 0.05 was considered significant.

## Results

This study was conducted on 138 patients with a mean age of 43.19 ± 11.3 years after laparoscopic cholecystectomy (Fig. 1). The majority of them were women (68.8%). Table 1 shows no difference between the demographic and clinical characteristics of the participants in the massage, TENS, and control groups (p > 0.05).

**Table 1:** Demographic and clinical characteristics of participants in massage, TENS, and control groups

Variables	Control	TENS	Massaging	P-value
sex				
male female	10(21.7) 36(78.3)	19(41.3) 27(58.7)	14(30.4) 32(69.6)	0.127*
Age	42.91 ± 11.50	42.07 ± 11.73	44.59 ± 10.91	0.559**
The history of abdominal surgery	25(54.3)	28(60.9)	20(43.5)	0.240*
Duration of surgery	54.67 ± 18.98	53.48 ± 18.99	54.46 ± 20.44	0.952**
Duration of anesthesia	72.93 ± 18.93	71.41 ± 18.45	71.95 ± 20.28	0.929**
Pethidine use (mg)	29.35 ± 10.93	27.17 ± 8.86	27.72 ± 9.47	0.543**

**Fig. 3:**
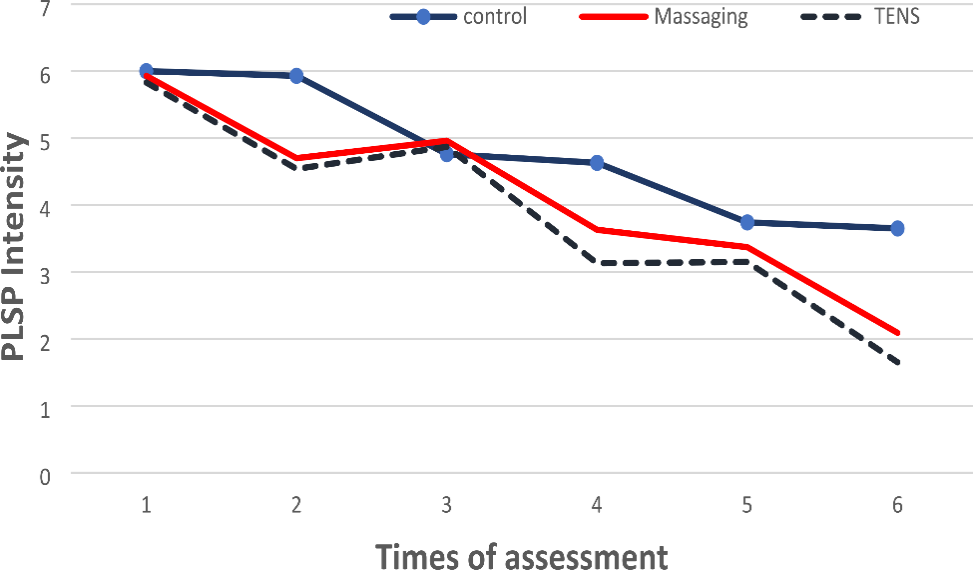
Mean scores of the shoulder pain severity after Cholecystectomy

The results of one-way analysis of variance are summarized in Table 2; Fig. 3. Levene’s test confirmed the assumption of homogeneity of variances (p > 0.05). Before the interventions, the results showed that the mean PLSP was not significantly different in the groups. However, after the interventions, the amount of PLSP in the massage therapy, TENS, and control groups decreased at all period times and was considered statistically significant (p < 0.001).

**Table 2:** Comparison of mean PLSP intensity scores in massaging, TENS, and control groups

Time	Control	TENS	Massaging	P-value*
4 h after surgery				
Before intervention After intervention	6.00 ± 1.86 5.93 ± 1.91	5.83 ± 1.78 4.54 ± 1.39	5.93 ± 1.97 4.70 ± 1.54	0.90 < 0.001
8 h after surgery				
Before intervention After intervention	4.76 ± 1.72 4.63 ± 1.81	4.87 ± 1.34 3.13 ± 1.42	4.96 ± 1.54 3.63 ± 1.61	0.83 < 0.001
12 h after surgery				
Before intervention After intervention	3.74 ± 1.54 3.65 ± 1.38	3.15 ± 1.19 1.65 ± 1.15	3.37 ± 1.52 2.09 ± 1.48	0.14 < 0.001
P-value**	< 0.001	< 0.001	< 0.001	

*ANOVA

** Repeated measure ANOVA

The mean difference in PLSP decrease in the three groups before and after the interventions were significant (p < 0.001) (Table 3).

**Table 3:** Comparison of the mean difference in shoulder pain intensity scores before and after the intervention in the massage, TENS, and control groups

Times	Groups	Mean ± SD	P-value*
1	Massaging TENS Control	1.23 ± 0.99 1.28 ± 1.02 0.06 ± 0.74	< 0.001
2	Massaging TENS Control	1.32 ± 0.79 1.73 ± 0.88 0.13 ± 1.04	< 0.001
3	Massaging TENS Control	1.28 ± 0.95 1.50 ± 1.14 0.08 ± 0.91	< 0.001

*one-way ANOVA

Comparing the two groups regarding changes in PLSP intensity showed that the effect of massage therapy and TENS was more significant than the control group (p < 0.001). However, there was no significant difference between the two interventions of massage therapy and TENS at any time (Table 4).

**Table 4:** Two-by-Two comparison groups regarding shoulder pain intensity at 4, 8, and 12 h after laparoscopic cholecystectomy

Times	Mean difference	P-value*
4 h after surgery		
Massaging-control	-1.17	< 0.001
control –TENS	-1.21	< 0.001
Massaging _TENS	-0.04	0.97
8 h after surgery		
Massaging-control	-1.19	< 0.001
control –TENS	-1.60	< 0.001
Massaging _TENS	-0.41	0.08
12 h after surgery		
Massaging-control	-1.19	< 0.001
control –TENS	-1.41	< 0.001
Massaging _TENS	-0.21	0.56

*Post hoc analysis, using Tukey’s method

In addition, Mauchly test confirmed the assumption of sphericity of the data. ANOVA with repeated measurements showed a significant difference between the three groups in the average intensity of PLSP after the intervention. Hence, the relief of pain in the intervention groups was more than the control group (p < 0.001) (Table 2).

## Discussion

The present study evaluated the effectiveness of classical hand massage in reducing PLSP compared to TENS. To our knowledge, no previous research compared these two interventions to relieve PLSP. Therefore, the current randomized trial was the first study following laparoscopic cholecystectomy. Based on our findings, TENS significantly reduced PLSP when added to multimodal treatment methods. In other studies on different types of surgery, the effect of active TENS has been compared to placebo [18, 20, 25], no treatment (Control) [17, 26] and pharmacological drugs [19, 27], so significant pain relief was identified in the active TENS group.

In a previous study, Asgari et al. [27] compared the effect of TENS with fentanyl on reducing PLSP in patients undergoing laparoscopic gynecologic surgery who had spinal anesthesia. They indicated that in the short-term evaluation, five minutes after fentanyl intervention, there was a more significant effect than TENS. Nevertheless, TENS was more effective after 30 min. Although their overall results showed no inferiority of fentanyl to TENS, both methods reduced PLSP.

In line with our study, Borges et al. [25] evaluated the effect of Active TENS and Placebo TENS compared to the control group on pain after laparoscopic cholecystectomy. The authors found that Active TENS for 30 min significantly reduced pain at the umbilical, subcostal, epigastric, and abdominal incision sites compared to placebo TENS and the control group. This study showed the superiority of Active TENS over placebo TENS and the control group that did not receive any intervention.

Also, Platon et al. [19] reported the effect of TENS on pain after laparoscopic gynecological surgery. One group received TENS and the other group received opioids; the results of this study showed significant pain relief in both groups after discharge from the recovery ward and hospital.

In another study, 52 patients undergoing inguinal hernia surgery were divided into two active TENS and control groups (26 people in each group) to investigate the effects of TENS on postoperative pain. The duration of the intervention was 24 h and applied five times after the operation for 30 min. In the experimental group, TENS was used with a frequency of 100 Hz and a pulse width of 100 microseconds. In the other group, TENS was applied without electrical stimulation. Similarly, the results indicated a significant reduction in pain in the Active TENS group [17].

However, in some studies, no evidence confirmed the effect of TENS on pain. In this regard, Kurata et al. [28] investigated the effects of TENS on pain after a cesarean section. In this clinical trial, 180 women were randomly divided into three groups: Active TENS, Placebo TENS and control. The results showed that the pain after the cesarean section was not significantly different between the groups. The findings of this study did not confirm our results. Differences in the target population may be the reason for this difference.

Also, in these three studies, no effect of TENS on pain at rest (static pain) after abdominal surgery [29], hysterectomy [30] and, hip fracture [31] was observed. The main reasons for this may be the difference in sample size, TENS application method and follow-up period. Although our findings were consistent only with the limited previous studies, the difference in surgical procedures and other factors influencing the perception of pain intensity should be considered.

We also found that classic hand massage significantly reduced PLSP. The present finding can improve our understanding of the value of massage therapy techniques on pain.

In a systematic review and meta-analysis, an evidence was presented that short-term or long-term massage is an effective method for reducing shoulder pain [32]. Consistent with our study, a case report by Zerkle and Gates (2020) examined the effect of 25 min of massage after laparoscopic abdominal surgery and reported that massage therapy significantly reduced PLSP [33]. Similarly, Sözen and Karabulut (2020) found that a 30-minute hand massage relieved pain after laparoscopic cholecystectomy [15].

In another study, 105 patients with rheumatoid arthritis were divided into three massage, reiki, and control groups to investigate the effects of hand massage and reiki on pain. The interventions were repeated six times and each time for 30 min. Similar to the current study, the results indicated a significant reduction in pain in both groups [34].

Rasooli et al. [14] used 15-minute hand massage techniques to relieve headaches after cesarean section and it was found that the VAS score decreased significantly in the hand massage group. Their results are consistent with the present findings.

In two clinical trials on patients undergoing heart surgery, the effect of hand massage on postoperative pain showed that in both studies, massage significantly reduced patients’ pain compared to those who received only routine care [35, 36].

In the present study, there was no significant difference in the dose of pethidine used between the three groups. Contrary to the current findings, some studies reported that massage and TENS reduced analgesic use compared to control or placebo groups [15, 37]. These differences can be attributed to differences in samples, pain intensity, methods, and study designs.

Several mechanisms have been proposed for massage therapy and TENS to relieve pain [38, 39]. According to the gate control theory, thick sensory fibers (A-β) stimulated by various factors such as massage and TENS are faster than thin fibers (A-δ and C) that cause pain transmission. These two modalities stimulate mechanoreceptors and thick sensory fibers and eventually slow down the transmission of pain signals to the brain. Also, by stimulating dense sensory fibers, the pain-inhibiting neurons are activated at the level of the dorsal horn. The pain is minimized by reducing the speed of the projection neurons [40]. In addition, it has been shown that massage therapy and TENS can effectively relieve pain by secreting endogenous opiates [38, 39].

### Limitations

Despite the strengths of the present study, our research has the following limitations:

1. Although the surgical procedure and anesthesia were the same for all patients, the patients underwent surgery under the supervision of different anesthesiologists and surgeons.
2. There was insufficient evidence on the best time for TENS and massage to start interventions to improve shoulder pain which may affect the results to somewhat.
3. Shoulder pain was assessed in all patients only at rest, and no separate pain measurement was performed at rest and in motion.
4. The primary source of patients’ pain (incision and shoulder pain) was not assessed when they requested analgesia.
5. The effect of interventions on patients’ shoulder pain was not assessed for a long time and was limited to the initial hours after surgery.
6. At the end, the sample size was small, and it was unclear whether the findings would be different if used in multiple centers in different environments and with larger sample sizes.

## Conclusion

It seems that massage therapy and TENS can be effective in PLSP relief. Considering that the shoulder pain is often expected after laparoscopy, and the large number of this clinical problem, specifies the need to pay more attention to this matter. It is recommended to use frequent massage therapy and TENS with a longer duration on patients’ having shoulder pain after other laparoscopic surgeries so that their effect on the amount of analgesia used by patients can be seen.

## Data Availability

The datasets used and/or analyzed during the current study available from the corresponding author on reasonable request.

## Declarations

All patients signed an informed consent form prior to their participation in the study.

## Abbreviations

TENS: Transcutaneous electrical nerve stimulation
PLSP: Post laparoscopic shoulder pain
VAS: Visual Analogue Scale
ASA: American Society of Anesthesiology

## References

[1] Di Buono G, Romano G, Galia M, Amato G, Maienza E, Vernuccio F (2021). Difficult laparoscopic cholecystectomy and preoperative predictive factors. Sci Rep.

[2] Kim HY, Choi JB, Min SK, Chang MY, Lim GM, Kim JE (2021). A randomized clinical trial on the effect of a lidocaine patch on shoulder pain relief in laparoscopic cholecystectomy. Sci Rep.

[3] Li XY, Tian M, Li AZ, Han CL, Li KZ (2021). The risk of shoulder pain after laparoscopic surgery for infertility is higher in thin patients. Sci Rep.

[4] Sao CH, Chan-Tiopianco M, Chung KC, Chen YJ, Horng HC, Lee WL (2019). Pain after laparoscopic surgery: focus on shoulder-tip pain after gynecological laparoscopic surgery. J Chin Med Association: JCMA.

[5] Hosseinzadeh F, Nasiri E, Behroozi T (2020). Investigating the effects of drainage by hemovac drain on shoulder pain after female laparoscopic surgery and comparison with deep breathing technique: a randomized clinical trial study. Surg Endosc.

[6] Abo Elfadl GM, Osman AM, Ghalyoom MF, Gad Al-Rab NA, Bahloul M. Preoperative duloxetine to prevent postoperative shoulder pain after gynecologic laparoscopy: a randomized controlled trial. Brazilian J Anesthesiology (Elsevier). 2021. 10.1016/j.bjane.2021.07.035 34411629

[7] Mirhosseini H, Avazbakhsh MH, Hosseini Amiri M, Entezari A, Bidaki R (2017). Effect of oral clonidine on Shoulder Tip Pain and hemodynamic response after laparoscopic cholecystectomy: a Randomized double blind study. Anesthesiology and pain Medicine.

[8] Rahimzadeh P, Faiz SHR, Latifi-Naibin K, Alimian M (2022). A comparison of effect of preemptive versus postoperative use of ultrasound-guided bilateral transversus abdominis plane (TAP) block on pain relief after laparoscopic cholecystectomy. Sci Rep.

[9] Komann M, Weinmann C, Schwenkglenks M, Meissner W (2019). Non-pharmacological methods and post-operative Pain Relief: an observational study. Anesthesiology and pain Medicine.

[10] Vickers A, Zollman C, Reinish JT (2001). Massage therapies. West J Med.

[11] Guo PP, Fan SL, Li P, Zhang XH, Liu N, Wang J (2020). The effectiveness of massage on peri-operative anxiety in adults: a meta-analysis of randomized controlled trials and controlled clinical trials. Complement Ther Clin Pract.

[12] Dreyer NE, Cutshall SM, Huebner M, Foss DM, Lovely JK, Bauer BA (2015). Effect of massage therapy on pain, anxiety, relaxation, and tension after colorectal surgery: a randomized study. Complement Ther Clin Pract.

[13] Chandrababu R, Nayak BS, Pai VB, George NR, Devi LS (2020). Effects of foot massage and patient education in patients undergoing coronary artery bypass graft surgery: a randomized controlled trial. Complement Ther Clin Pract.

[14] Rasooli AS, Atashkhoei S, Ghahramanian A, Goljaryan S, Zarie L (2018). The Effect of Head-Neck and Hand Massage on spinal Headache after Cesarean Section: Randomized Clinical Trial. J Res Med Dent Sci.

[15] Sözen KK, Karabulut N (2020). Efficacy of Hand and Foot Massage in anxiety and Pain Management following laparoscopic cholecystectomy: a controlled Randomized Study. Surgical laparoscopy. Endoscopy & Percutaneous Techniques.

[16] Visconti MJ, Haidari W, Feldman SR (2020). Transcutaneous electrical nerve stimulation (TENS): a review of applications in dermatology. J Dermatolog Treat.

[17] Yılmaz E, Karakaya E, Baydur H, Tekin İ (2019). Effect of Transcutaneous Electrical nerve stimulation on Postoperative Pain and patient satisfaction. Pain Manage Nursing: Official J Am Soc Pain Manage Nurses.

[18] Mahure SA, Rokito AS, Kwon YW (2017). Transcutaneous electrical nerve stimulation for postoperative pain relief after arthroscopic rotator cuff repair: a prospective double-blinded randomized trial. J Shoulder Elbow Surg.

[19] Platon B, Mannheimer C, Andréll P (2018). Effects of high-frequency, high-intensity transcutaneous electrical nerve stimulation versus intravenous opioids for pain relief after gynecologic laparoscopic surgery: a randomized controlled study. Korean J Anesthesiology.

[20] da Silva MP, Liebano RE, Rodrigues VA, Abla LE, Ferreira LM (2015). Transcutaneous electrical nerve stimulation for pain relief after liposuction: a randomized controlled trial. Aesthetic Plast Surg.

[21] Park SJ (2020). Postoperative shoulder pain after laparoscopic surgery. J Minim Invasive Surg.

[22] Adlan ASA, Azhary JMK, Tarmidzi HZM, Kamarudin M, Lim RCS, Ng DSW (2022). Post Laparoscopy Pain Reduction Project I (POLYPREP I): intraperitoneal normal saline instillation—a randomised controlled trial. BMC Womens Health.

[23] Alghadir AH, Anwer S, Iqbal A, Iqbal ZA (2018). Test-retest reliability, validity, and minimum detectable change of visual analog, numerical rating, and verbal rating scales for measurement of osteoarthritic knee pain. J pain Res.

[24] Saatsaz S, Rezaei R, Alipour A, Beheshti Z (2016). Massage as adjuvant therapy in the management of post-cesarean pain and anxiety: a randomized clinical trial. Complement Ther Clin Pract.

[25] Borges MR, de Oliveira NML, Antonelli IBS, Silva MB, Crema E, Fernandes L (2020). Transcutaneous electrical nerve stimulation is superior than placebo and control for postoperative pain relief. Pain Manage.

[26] Kasapoğlu I, Kasapoğlu Aksoy M, Çetinkaya Demir B, Altan L (2020). The efficacy of transcutaneous electrical nerve stimulation therapy in pain control after cesarean section delivery associated with uterine contractions and abdominal incision. Turkish J Phys Med Rehabilitation.

[27] Asgari Z, Tavoli Z, Hosseini R, Nataj M, Tabatabaei F, Dehghanizadeh F (2018). A comparative study between Transcutaneous Electrical nerve stimulation and fentanyl to relieve Shoulder Pain during laparoscopic gynecologic surgery under spinal anesthesia: a Randomized Clinical Trail. Pain Res Manage.

[28] Kurata NB, Ghatnekar RJ, Mercer E, Chin JM, Kaneshiro B, Yamasato KS (2022). Transcutaneous Electrical nerve stimulation for Post-Cesarean Birth Pain Control: a Randomized Controlled Trial. Obstet Gynecol.

[29] Rakel B, Frantz R (2003). Effectiveness of transcutaneous electrical nerve stimulation on postoperative pain with movement. J pain.

[30] Karaman S, Karaman T, Deveci H, Ozsoy AZ, Delibas IB (2021). Effect of transcutaneous electrical nerve stimulation on quality of recovery and pain after abdominal hysterectomy. J Anaesthesiol Clin Pharmacol.

[31] Elboim-Gabyzon M, Andrawus Najjar S, Shtarker H (2019). Effects of transcutaneous electrical nerve stimulation (TENS) on acute postoperative pain intensity and mobility after hip fracture: a double-blinded, randomized trial. Clin Interv Aging.

[32] Yeun YR (2017). Effectiveness of massage therapy for shoulder pain: a systematic review and meta-analysis. J Phys Therapy Sci.

[33] Zerkle D, Gates E (2020). The Use of Massage Therapy as a Nonpharmacological Approach to Relieve Postlaparoscopic Shoulder Pain: a Pediatric Case Report. Int J Therapeutic Massage Bodyw.

[34] SevgiÜnal Aslan K, Çetinkaya F (2023). The effects of Reiki and hand massage on pain and fatigue in patients with rheumatoid arthritis. EXPLORE.

[35] Boitor M, Martorella G, Maheu C, Laizner AM, Gélinas C. Effects of Massage in reducing the Pain and anxiety of the cardiac surgery critically Ill-a Randomized Controlled Trial. Pain medicine (Malden, Mass). 2018;19(12):2556–69. 10.1093/pm/pny055 29618079

[36] Braun LA, Stanguts C, Casanelia L, Spitzer O, Paul E, Vardaxis NJ (2012). Massage therapy for cardiac surgery patients–a randomized trial. J Thorac Cardiovasc Surg.

[37] Parseliunas A, Paskauskas S, Kubiliute E, Vaitekunas J, Venskutonis D (2021). Transcutaneous Electric nerve stimulation reduces Acute Postoperative Pain and Analgesic Use after Open Inguinal Hernia surgery: a Randomized, Double-Blind, placebo-controlled trial. J pain.

[38] Engen DJ, Carns PE, Allen MS, Bauer BA, Loehrer LL, Cha SS (2016). Evaluating efficacy and feasibility of transcutaneous electrical nerve stimulation for postoperative pain after video-assisted thoracoscopic surgery: a randomized pilot trial. Complement Ther Clin Pract.

[39] Sahraei F, Rahemi Z, Sadat Z, Zamani B, Ajorpaz NM, Afshar M (2022). The effect of swedish massage on pain in rheumatoid arthritis patients: a randomized controlled trial. Complement Ther Clin Pract.

[40] Mokhtari T, Ren Q, Li N, Wang F, Bi Y, Hu L (2020). Transcutaneous Electrical nerve stimulation in relieving Neuropathic Pain: Basic Mechanisms and clinical applications. Curr Pain Headache Rep.

